# Associations Between ADHD Symptoms and Maternal and Birth Outcomes: An Exploratory Analysis in a Multi-Country Cohort of Expectant Mothers

**DOI:** 10.1177/10870547221105064

**Published:** 2022-07-11

**Authors:** Aja Louise Murray, Diana Taut, Adriana Baban, Chad Lance Hemady, Susan Walker, Joseph Osafo, Siham Sikander, Mark Tomlinson, Stefani Du Toit, Marguerite Marlow, Catherine L. Ward, Asvini Fernando, Bernadette Madrid, Vo Van Thang, Hoang Dinh Tuyen, Michael Dunne, Claire Hughes, Pasco Fearon, Sara Valdebenito, Manuel Eisner

**Affiliations:** 1 University of Edinburgh, UK; 2 Babes-Bolyai University, Cluj-Napoca, Romania; 3 The University of the West Indies, Kingston, Jamaica; 4 University of Ghana, Accra, Ghana; 5 University of Liverpool, UK; 6 Stellenbosch Uni`versity, Cape Town, South Africa; 7 Queens University, Belfast, UK; 8 University of Cape Town, South Africa; 9 University of Kelaniya, Sri Lanka; 10 University of the Philippines, Manila, Philippines; 11 Hue University, Vietnam; 12 Queensland University of Technology, Brisbane, Australia; 13 University of Cambridge, UK; 14 University College London, UK; 15 University of Zurich, Switzerland

**Keywords:** ADHD symptoms, perinatal, depression, stress, social support, substance use

## Abstract

**Objective:**

ADHD symptoms can adversely impact functioning in a range of domains relevant for maternal well-being and fetal development; however, there has been almost no research examining their impact during pregnancy. We aimed to address this gap.

**Method:**

We used data (*n* = 1,204) from a longitudinal birth cohort study spanning eight countries to address this gap.

**Results:**

ADHD symptoms in the third trimester of pregnancy were associated with lower social support from family (*b* = −0.16, *p* = .031), friends (*b* = −0.16, *p* = .024), and significant others (*b* = −0.09, *p* = .001); higher stress (*b* = 0.34, *p* < .001) and depressive symptoms (*b* = 0.31, *p* < .001), and increased likelihood of an unwanted pregnancy (*b* = 0.30, *p* = .009). Significant associations with tobacco use (*b* = 0.36, *p* = .023) and premature birth (*b* = 0.35, *p* = .007) did not survive correction for multiple comparisons and there were no significant associations with alcohol use, low birth weight, or unplanned pregnancy.

**Conclusion:**

Results suggest that women with ADHD symptoms could benefit from earlier, more regular screening for mental health difficulties and greater mental health support during pregnancy.

ADHD is characterized by the core symptoms of inattention (e.g., difficulty concentrating on a task for a sustained period) and/or hyperactivity/impulsivity (e.g., difficulty sitting still and internal feelings of restlessness), occurring with a frequency and severity that is clinically impairing (American Psychiatric Association, 2013). These symptoms sit on a continuum, with meaningful etiological and phenotypic variation both above and below clinical thresholds (Groen-Blokhuis et al., 2014). Accordingly, impairments and risks associated with ADHD symptoms extend into the non-clinical range and include difficulties in educational, occupational, and social functioning; increased risks of accidents and unintentional injuries; and mental health, behavioral, and substance use issues (e.g., Fite et al., 2014; Murray et al., 2017, 2018; Oliver et al., 2015; Ruiz-Goikoetxea et al., 2018).

While traditionally perceived as primarily affecting children, especially young boys, ADHD symptoms are now also recognized to affect both adult men and women, with an attenuated gender gap in those with a diagnosis in adulthood relative to childhood. Estimates suggest that approximately 3% of adult women meet diagnostic criteria for ADHD, with a male : female ratio of only 1:1 to 2:1 (Kessler et al., 2005; Williamson & Johnston, 2015). However, both persisting perceptions of ADHD as a “male disorder” and gender differences in manifestation and comorbidities mean that women with ADHD symptoms are likely to be under-diagnosed (Gershon & Gershon, 2002; Williamson & Johnston, 2015). Thus, while a non-trivial proportion of women enter pregnancy with symptoms—including sub-clinical symptoms—of ADHD, many of these women will not have had their symptoms recognized prior to pregnancy.

ADHD symptoms can be impairing across the entire life cycle, but may have additional significance in the perinatal period, as their sequelae could impact fetal development. ADHD symptoms have, for example, been associated with an increased risk of sexual risk-taking leading to a greater prevalence of unplanned pregnancies (and it is presumed, by extension, of unwanted pregnancies; Ninowski et al., 2007; Owens & Hinshaw, 2020). Unplanned pregnancies are in turn associated with higher levels of perinatal maternal depressive symptoms; poorer health behaviors prior to and during pregnancy, such as a lack of pre-pregnancy vitamin supplementation, smoking during pregnancy, and lower attendance at antenatal appointments; and poorer maternal-fetal attachment (Abajobir et al., 2016; Goossens et al., 2016; Yanikkerem et al., 2013). There is also mixed evidence that unplanned pregnancies are associated with an increased risk of poorer birth outcomes, such as babies being born preterm and with lower birth weight (Flower et al., 2013; Goossens et al., 2016). Taken together, these findings raise the possibility that women with ADHD symptoms are at greater risk of pregnancy outcomes such as low birth weight or premature infants.

During pregnancy, mental health and wellbeing issues commonly associated with ADHD symptoms, such as anxiety, depression, and stress (Biederman et al., 2008; Krone & Newcorn, 2015; Murray et al., 2022) may also contribute to an increased risk of poorer fetal and child developmental outcomes. Previous research suggests that these factors can affect prenatal development via mechanisms such as increased fetal exposure to glucocorticoids, which can affect the methylation status of genomic sites relevant for child hypothalamic-pituitary (HPA) axis functioning (Bale, 2015; Sosnowski et al., 2018). In the longer term, prenatal stress exposure has been linked to child outcomes such as lower intellectual functioning and emotional and behavioral problems (Bergman et al., 2007; Betts et al., 2014; Laplante et al., 2004; Martinez-Torteya et al., 2016). Further, the psychological and behavioral symptoms of maternal distress, including diminished maternal capacity to engage in fetal bonding and to make healthy choices may affect fetal and child development (Schmidt et al., 2016; Vythilingum et al., 2012).

ADHD symptoms may also make it more difficult for women to adhere to health advice during pregnancy. For example, Ninowski et al., (2007) proposed that difficulties associated with ADHD symptoms, such as ineffective decision-making, forgetfulness and distractibility, and poor organizational and time management skills, could compromise expectant mothers’ ability to follow nutritional and physical activity guidelines and attend the recommended number of antenatal appointments. Substance use is a health behavior of particular significance in relation to ADHD symptoms. Previous research has identified associations between ADHD symptoms and substance use (Lee et al., 2011; Murray et al., 2017), suggesting that the offspring of expectant mothers with ADHD could be at elevated risk of being exposed to harmful levels of teratogens in utero. The risks of alcohol use during pregnancy are particularly well-established, with prenatal alcohol exposure representing the most significant preventable cause of intellectual disability (Committee on Substance Abuse and Committee on Children with Disabilities, 2000). There is also evidence that cigarette smoking and other commonly available substances such as over-counter analgesics may have an adverse impact on fetal neurodevelopment (Gou et al., 2019; Knopik et al., 2016). It is, therefore, important to establish whether the links between ADHD symptoms and substance use extend to the antenatal period, increasing the risk of poorer fetal developmental outcomes.

Finally, mental and physical health correlates of ADHD symptoms during pregnancy may occur in the context of interpersonal relationship issues (Ninowski et al., 2007). For example, ADHD symptoms have been associated with poorer intimate partner relationship quality, increased intimate partner violence victimization and perpetration, and a higher likelihood of divorce (Biederman et al., 2006; Bruner et al., 2015; Eddy et al., 2019; Wymbs et al., 2017), suggesting that women with ADHD symptoms may be less able to draw on partner support during pregnancy. ADHD symptoms in adulthood have also been associated with poorer friendship quality (e.g., McKee, 2017), such that broader social support may also be reduced for pregnant women with ADHD symptoms. Although the empirical evidence is somewhat mixed, partner, and social support during pregnancy have been proposed to help reduce stress and mental health issues, or mitigate their impact (Giesbrecht et al., 2013; Hetherington et al., 2015; Rini et al., 2006). Thus, pregnant women with ADHD symptoms may be lacking an important buffering factor with respect to their and their fetus’ health and wellbeing.

Despite the potential additional risks for women with ADHD symptoms in the perinatal period, this topic has, with a few notable exceptions (Andersson et al., 2020; A. S. Baker et al., 2020; Eddy et al., 2019; Jones et al., 2018; Ninowski et al., 2007), attracted very little direct research (see Kittel-Schneider et al., 2021 for a review). In one study of 86 expectant mothers in Canada, Ninowski et al. (2007) found that ADHD symptoms were associated with elevated anxiety and depression, less positive parental expectations, lower maternal self-efficacy, and lower attendance to prenatal check-ups, but were unrelated to poor health behaviors such as physical inactivity, unhealthy eating patterns, smoking, and alcohol consumption. However, a more recent and larger study of 198 women in the United States found that ADHD symptoms were associated with poorer health behaviors during pregnancy (Jones et al., 2018). Likewise, in another USA-based study of the impact of inattention, hyperactivity, and impulsivity on professional life, daily life, and relationship impairment in 250 expectant mothers, Eddy et al. (2019) found that inattention symptoms (but not hyperactivity) predicted poorer functioning in all three domains and impulsivity predicted poorer functioning in professional life and relationship impairment. Finally, a large medical register-based study in Sweden and Norway found that women with ADHD were more likely to smoke in both early and late pregnancy; this association held after adjusting for maternal education, year of child’s birth, and psychiatric comorbidities (Andersson et al., 2020).

Importantly, all of the studies of the impact of maternal ADHD symptoms in pregnancy identified in Kittel-Schneider et al.’s (2021) systematic review involved samples from high income countries. Currently, we know almost nothing about the impact of ADHD symptoms in lower resource settings. We do, however, know from epidemiological studies that adulthood ADHD is an issue in low- and middle-income countries, where it is strongly associated with outcomes such as anxiety, depression, behavioral disorders, substance use, and impairments in domains such as cognition and social interaction (Fayyad et al., 2017). It is, however, also under-recognized and under-treated in these countries (Fayyad et al., 2017). Taken together, there is a strong need for better information about the impact of ADHD symptoms in pregnancy, especially in low- and middle-income countries where there is next to no evidence currently available.

An improved understanding of the impacts of ADHD symptoms in pregnancy offers a range potential of clinical implications. For example, evaluating the relative risks and benefits of pharmacological treatments for ADHD symptoms during pregnancy requires evidence regarding the possible range and severity of the impacts of ADHD symptoms in pregnancy (Freeman, 2014). For women with known ADHD symptoms, understanding the primary effects of ADHD symptoms during pregnancy can also inform screening and referral pathways to better support maternal well-being and fetal health. Further, if the impacts of ADHD symptoms on maternal and fetal health during pregnancy prove sufficiently common and severe, it may be helpful to screen for ADHD symptoms in pregnancy.

We thus used data from an eight-country birth cohort study to examine the associations between ADHD symptoms in the third trimester of pregnancy and a range of maternal and birth outcomes based on previous evidence of ADHD sequelae likely to be especially relevant in the perinatal period. Specifically, we examined the relations between ADHD symptoms during pregnancy and social support, substance use, stress, depressive symptoms, unplanned, and unwanted pregnancy as reported in the third trimester of pregnancy and with infant low birth weight and prematurity.

## Method

### Participants

Participants were from the Evidence for Better Lives Study—Foundational Research (EBLS-FR) study (Valdebenito et al., 2020). Participants were 1,204 pregnant women in their third trimester of pregnancy (a small number of twin pregnancies were excluded) from eight sites selected as medium-sized cities/conurbations in a low- or middle-income country (analysis-specific Ns are provided in Table 2). The sites, chosen with the goal of sampling a diversity of cultural, political, and social contexts, were: Valenzuela in the Philippines; Hue in Vietnam; Ragama in Sri Lanka; Tarlai Kalan in Pakistan; Cluj-Napoca in Romania; Worcester in South Africa; Koforidua in Ghana; and Kingston in Jamaica. The study thus includes at least one country from each major world region as defined by the World Health Organization (WHO). Within each site, pregnant women were recruited through primary health clinics during their standard antenatal care visits. Women were eligible to take part in the study if they were in the third trimester of pregnancy (29–40 weeks of gestation), aged over 18, had their main residence within the defined geographical regions of the EBLS-FR site in which they were recruited, and were able to provide informed consent. No additional exclusion criteria were applied. Table 1 provides further participant characteristic information.

**Table 1.** Participant Characteristics and Descriptive Statistics.

	*N*	*M*	*SD*
Demographic information			
Age	1,204	28.26	5.79
Perceived social status	1,191	5.23	2.03
Country	1,204	Ghana = 150, Jamaica = 152, Pakistan = 147, Philippines = 154, Romania = 150, South Africa = 150, Sri Lanka = 152, and Vietnam = 149	
ADHD symptoms			
ADHD—concentration problems	1,189	1.99	1.03
ADHD—dependent on others	1,191	1.83	1.03
ADHD—procrastination	1,192	2.35	1.15
ADHD—trouble relaxing	1,191	2.34	1.24
ADHD—difficulty staying seated	1,188	1.86	1.01
ADHD mean symptoms score	1,193	2.08	0.68
Perinatal outcomes			
Social support—family	1,195	4.06	0.92
Social support—friends	1,194	3.53	1.15
Social support—significant other	1,194	4.17	0.86
Substance use—tobacco	1,181	No = 1051, yes = 130	
Substance use—alcohol	1,185	No = 1011, yes = 174	
Depressive symptoms	1,196	0.76	0.53
Stress	1,195	2.03	0.57
Unwanted pregnancy	1,199	Wanted = 887, unwanted = 312	
Unplanned pregnancy	1,202	Planned = 612, unplanned = 590	
Low birth weight^a^	1,011	Low birth weight = 79, normal birth weight = 932	
Prematurity^b^	1,043	Premature = 69, term = 974	

*Note*. SES = socioeconomic status based on the 10-point MacArthur Scale of Subjective Social Status, with higher scores representing higher perceived status (N. E. Adler et al., 2000).

a The mean birth weight was 3.11 kg (*SD* = 0.48), with a minimum birth weight of 1 kg and a maximum birth weight of 5 kg.

b The mean age at gestational age was 38.9 weeks (*SD* = 1.78), with a minimum of 25 weeks and a maximum of 43 weeks. The possible score ranges for the questionnaire measures were as follows: Perceived social status = 1 to 10; ADHD item scores = 1 to 5; ADHD mean scores = 1 to 5; Social support—family = 1 to 5; Social support—friends = 1 to 5; Social support—significant other = 1 to 5; Depressive symptoms = 0 to 3; Stress = 1 to 4.

**Table 2.** Fixed Effects From Mixed Effects Models of Effect of ADHD Symptoms on Maternal and Birth Outcomes.

Outcome	*N*	*B*	*SE*	*p*-Value
Prenatal social support—family	1,192	−0.16	0.060	.031
Prenatal social support—friends	1,191	−0.16	0.058	.024
Prenatal social support—significant other^a^	1,191	−0.09	0.038	.001
Prenatal substance use—tobacco ^a^	1,180	0.36	0.160	.023
Prenatal substance use—alcohol	1,184	0.28	0.281	.317
Prenatal depressive symptoms	1,193	0.30	0.021	<.001
Prenatal stress	1,193	0.34	0.029	<.001
Unwanted pregnancy	1,190	0.30	0.116	.009
Unplanned pregnancy ^a^	1,193	−0.12	0.098	.213
Low birth weight	1,006	0.42	0.240	.080
Prematurity^a^	1,044	0.35	0.193	.007

*Note*. Full output available at: https://osf.io/jydm8/?view_only=5e9cf62150c24f68bcc8f5f2eb4624db. Correction for multiple comparisons yields an adjusted alpha threshold of .0045

a Convergence failures in the maximal random effects structures model necessitated the removal of the random slope effect. Results are based on the random intercepts model.

### Procedure

Data were collected in two waves, the first in the third trimester of pregnancy and the second between 3 and 39 weeks after the birth of the child (*M* = 14, *SD* = 5.3). Data at the third trimester were collected by trained fieldworkers using computer-assisted personal interviews (CAPI). Participants were interviewed in the antenatal clinics where they attended their healthcare appointment in a private space. At this stage participants reported on their health, wellbeing, adversity exposures, feelings about their pregnancy, reproductive history, attitudes toward future parenting, and social support. Following the birth of the child, participants were re-contacted to provide follow-up information. At this stage participants provided information about the birth of the child, early child health, and their current wellbeing. Further information on the study can be found in the study protocol paper (Valdebenito et al., 2020).

### Ethics

Prior to data collection, the study was approved by the ethics committees of the University of Cambridge and the Universities/research institutes in each country. Written informed consent was collected from all participants. All procedures complied with the Helsinki Declaration of 1975, as revised in 2008.

### Measures

All measures were translated from English (which served as the source language) into nine other languages: Urdu (Pakistan), Afrikaans and IsiXhosa (South Africa), Romanian (Romania), Filipino (Tagalog; the Philippines), Sinhala and Tamil (Sri Lanka), Vietnamese (Vietnam), and Twi (Ghana). The English versions were used in Jamaica. These languages were selected based on reflecting the largest linguistic groups in each site. The translation process was guided by best practice guidelines offered by the World Health Organization and the Translation Review Adjudication Pretest Documentation (TRAPD) method which is considered state of the art: https://europeanvaluesstudy.eu/methodology-data-documentation/survey-2017/methodology/the-trapd-method-for-survey-translation/. Specifically, two independent translators produced forward translations which were then harmonized. Fieldworkers were also provided with training to equip them to address any ambiguities or misunderstandings. All measures were pre-tested using a convenience sample of between 5 and 10 women in each site. These women were selected to be of similar background to the target participants. Minor corrections were made based on the results of the pre-testing.

### ADHD Symptoms

ADHD symptoms were measured using five items in the questionnaire administered during the third trimester of pregnancy. Three items were adapted from the Adult ADHD Self-report Scale (ASRS; L. A. Adler et al., 2006) for adult ADHD symptoms and an additional two items were adapted from the age 20 wave of the Zurich Project on Social Development from Childhood to Adulthood (*z*-proso Eisner et al., 2018). This latter instrument was itself an adult adaptation of the self-report Social Behavior Questionnaire (SBQ; Tremblay et al., 1991). These items were selected based on their applicability to adult women, given the criticism of many ADHD measures as being less well-calibrated to female ADHD symptoms (e.g., Williamson & Johnston, 2015). Responses were recorded on a five-point Likert-type scale from 1 = Never to 5 = Always. Item scores were averaged to provide a composite score with higher scores representing higher levels of ADHD symptoms. Cronbach’s alpha for these scores was .59. Item wordings in English are provided in Supplemental Material.

### Social Support

Social support was measured using the self-reported Multidimensional Scale of Perceived Social Support (Zimet et al., 1988, 1990), administered during the third trimester of pregnancy. The scale measures social support received from three sources: family, friends, and a significant other in three subscales (four items per subscale). In the version used in EBLS, responses are recorded on a five-point Likert-type scale from 1 = Strongly disagree to 5 = Strongly Agree. Item scores were averaged to provide composite scores for the family, friends, and significant others, with higher scores representing higher levels of social support from a given source. Separate domain scores were used rather than a general social support score because the correlations between the different domains were only moderate (*r* = .42–.61). Cronbach’s alphas for the family, friends, and significant other subscale scores were: .87, .92, and .85 respectively

### Substance Use

Substance use was measured using the self-reported Alcohol, Smoking, and Substance Use Involvement Screening Test (ASSIST; WHO ASSIST Working Group, 2002), administered during the third trimester of pregnancy. The instrument measures previous 6-month prevalence of tobacco, alcohol, cannabis, cocaine, amphetamine, sedative, hallucinogen, opioid, and inhalant use. Substance use was recorded on a 5-point Likert-type scale from 1 = never to 5 = daily or almost daily and recoded into “yes” = 1 versus “no” = 0 to distinguish women who never used substances from those who used them at all In the current study we focus on the alcohol and tobacco use items given their higher prevalence and previous evidence of associations with adverse impacts on fetal development.

### Depressive Symptoms

The PHQ-9 measures depressive symptoms experienced in the previous fortnight. Its nine items are based on DSM-IV-TR criteria for depression and include the following symptoms: anhedonia, dysphoria, sleep disturbances, fatigue, changes in eating, low self-esteem, concentration difficulties, hypo- or hyper-active behaviors, and thoughts of suicide or self-harm (Kroenke et al., 2001). Responses were recorded on a 4-point Likert-type scale from 0 = not at all to 3 = nearly every day. Composite scores were derived as the mean of item responses with higher scores representing higher depressive symptom levels. Cronbach’s alpha for this scale was .76.

### Stress

Stress was measured using the *Perceived Stress Scale* (PSS: Cohen, 1988). The PSS is a 10-item measure capturing the extent to which participants felt under stress in the previous month. Item responses were recorded on a 4-point Likert-type scale from 1 = not at all to 4 = nearly every day and averaged to provide an overall stress composite score. Higher scores indicate higher levels of stress. Cronbach’s alpha for these scores was .76.

### Unwanted Pregnancy

Unwanted pregnancy was measured using a single self-reported item in the baseline wave: “Was this an unwanted pregnancy.” Respondents were offered a binary *yes* = 1 or *no* = 0 response scale. The item was adapted from the *South Asian Birth Cohort* (*START*) prenatal questionnaire (Anand et al., 2013).

### Planned Pregnancy

Planned pregnancy was measured using a single self-report item in the baseline wave “Did you plan your pregnancy?” Respondents were offered a binary *yes* = 1 or *no* = 0 response scale. The item was adapted from the *South Asian Birth Cohort* (*START*) prenatal questionnaire (Anand et al., 2013).

### Low Birth Weight and Prematurity

Birth weight information was collected at the follow-up wave from varying available sources, including the child’s health passport and maternal self-reports. In some sites, where possible, missing data were followed up via the local health facility where the child was born. Low birth weight was defined using the standard definition of being born with a weight of <2,500 g. Gestational age at birth (in weeks) was collected at the follow-up wave, drawing on the same sources. Prematurity was defined as being born before 37 weeks of gestation.

### Statistical Procedure

Multi-level linear and logistic regression models were fit with linear or logistic link functions for continuous and binary outcomes respectively, with separate models estimated for each outcome. We estimated only unadjusted models (i.e., no covariates were included) to obtain estimates of the raw associations between ADHD symptoms and maternal and birth outcomes. Models were estimated using maximum likelihood estimation in the lme4 package in R statistical software. Random intercepts and random slopes were both included to account for the fact that our sampling design involved eight clusters (countries). Given that there were very few missing data for most models, missingness was dealt with using listwise deletion. Table 2 presents Ns utilized in each model. The statistical significance of the fixed effects were evaluated using the Satterthwaite method to estimate degrees of freedom (see, e.g., Luke, 2017), drawing on the lmerTest package in R statistical software (Kuznetsova et al., 2017). Given that multiple outcomes were examined, it is also helpful to consider statistical significance in the context of a correction for multiple comparisons. Using the conservative Bonferroni correction, an adjusted alpha threshold for these analyses would be .0045.

## Results

Table 1 provides descriptive statistics for the ADHD symptoms and outcome measure variables. Reflecting the normative nature of the sample, average symptom frequencies were generally in the “never” to “rarely” response option range. The most common ADHD symptoms were procrastination and having difficulty unwinding or relaxing when having time to oneself.

Table 2 provides fixed effect parameters for the effect of ADHD symptoms on a range of hypothesized perinatal outcomes. Full output i.e., the full fixed and random effect parameters can be found at: https://osf.io/jydm8/?view_only=5e9cf62150c24f68bcc8f5f2eb4624db. Analyses suggested that ADHD symptoms are significantly related to lower social support from family (*b* = −0.16, *p* = .031), friends (*b* = −0.16, *p* = .024), and significant others (*b* = −0.09, *p* = .001); greater tobacco use (*b* = 0.36, *p* = .023); and higher levels of depressive symptoms (*b* = 0.30, *p* < .001) and stress (*b* = 0.34, *p* < .001) during pregnancy. ADHD symptoms were also associated with a greater likelihood of the pregnancy being unwanted (*b* = 0.30, *p* = .009) and with premature birth (*b* = 0.35, *p* = .007). However, after correcting for multiple comparisons, only support from significant others, stress, and depression remained statistically significant.

ADHD symptoms were not significantly related to alcohol use during pregnancy nor to the pregnancy being unplanned, nor to low birth weight. Some caution is due, however, in the interpretation of the significant effects on tobacco use, social support from significant other, unplanned pregnancy, and low birth weights because it was necessary to trim the random effects structure to random intercepts only to achieve convergence. Specifically, for these, a random slopes model could not be fit and a random intercepts model was fit instead. The standard errors for these effects may, therefore, be slightly smaller than they should be.

## Discussion

Despite a long-dominant conceptualization of ADHD as a problem primarily affecting young boys, there is gradually increasing recognition that ADHD symptoms affect adult women (Williamson & Johnston, 2015). Indeed, estimates suggest that 3% of women may experience clinically significant ADHD symptoms in adulthood; a rate estimated to give a male : female gender ratio of only 2:1 to 1:1 (Kessler et al., 2005; Williamson & Johnston, 2015). There has, however, been very little research examining the impact of ADHD symptoms on pregnant women and their offspring, even though ADHD symptoms are known to impact domains of functioning that are of core relevance to a healthy perinatal period. Using data from an eight-country birth cohort, we explored the associations between ADHD symptoms in pregnancy and a range of perinatal correlates. ADHD symptoms were related to lower levels of social support from family, friends, and significant others; to increased tobacco (but not alcohol) use; to higher levels of stress and depression during pregnancy; and to premature birth. However, the associations with tobacco use and premature birth did not survive a Bonferroni correction for multiple comparisons. ADHD symptoms were also associated with an increased likelihood that a pregnancy was unwanted but not with a pregnancy being unplanned. There were no associations between ADHD symptoms and giving birth to a low-birth-weight infant.

The associations with stress and depressive symptoms are consistent with previous research suggesting that ADHD symptoms often co-occur with internalizing problems such as anxiety and depression (Jarrett, 2016; Kessler et al., 2006; Murray et al., 2020), including in pregnancy (Ninowski et al., 2007). These associations probably reflect a combination of mechanisms, including the emotional dysregulation difficulties that are a common feature of ADHD and the effects of encountering challenges in occupational and relationship functioning associated with ADHD symptoms (Murray et al., 2020). The additional demands of pregnancy may further exacerbate these difficulties (Eddy et al., 2019; Freeman, 2014).

The associations identified with stress and depression suggest that women in antenatal care with known ADHD symptoms could benefit from earlier and more frequent screening for internalizing symptoms in order to provide timely intervention and minimize their impact on mother and child. However, ADHD symptoms are more likely to be missed in females than in males, so many women may not have had their symptoms recognized previously (Williamson & Johnston, 2015). In fact, some evidence suggests that these women may first be identified when presenting not with ADHD symptoms but with internalizing difficulties. As such, in countries where depression screening is already part of routine antenatal care, it may be beneficial to include brief follow-up ADHD screens for those who screen positive for depression. Doing so could provide an opportunity to address the potential under-identification of women with ADHD symptoms at a critical juncture.

Though the evidence was less compelling given that the effect did not survive Bonferroni correction for multiple comparisons, ADHD symptoms were also associated with increased tobacco use during pregnancy. This is consistent with previous evidence that ADHD symptoms are related to greater cigarette use and dependence (Rohde et al., 2004; Treur et al., 2019). Taken together with our findings and previous medical register-based research, it seems likely that women with ADHD symptoms may experience somewhat greater difficulties in reducing their tobacco use during pregnancy and could benefit from cessation support (Andersson et al., 2020). We did not replicate previous associations between ADHD symptoms and alcohol use in adulthood (e.g., L. Baker et al., 2012). This lack of association may; however, reflect the inclusion of study sites where alcohol use among women is rare, resulting in a suppression of variance in maternal alcohol use related to ADHD symptoms. However, one other small previous study also found no association between ADHD symptoms and either alcohol or cigarette use during pregnancy (Ninowski et al., 2007). Further research will be required to clarify the extent of association between ADHD symptoms and substance use during pregnancy.

Our study also identified a potential association between ADHD symptoms and unwanted pregnancy but not unplanned pregnancy. The association with unwanted pregnancy is consistent with previous evidence that ADHD symptoms are associated with a greater risk of unintended pregnancies (Owens & Hinshaw, 2020). Previous research has suggested that ADHD symptoms are associated with greater sexual risk-taking, which may account for the association with unwanted pregnancies (Owens & Hinshaw, 2020). However, ADHD symptoms are also associated with a greater likelihood of involvement in relationships affected by intimate partner violence (e.g., Wymbs et al., 2017), therefore, it is also possible that women with ADHD symptoms are more likely to experience sexual coercion leading to unwanted pregnancies. Similarly, for women in relationships in which they hold little decision-making power, pregnancies may be planned and yet still unwanted by the female partner. The converse may also be more frequently true in contexts where pregnancy planning is uncommon; namely, that a pregnancy is not planned and yet not unwanted. This could also contribute to the divergent association seen between ADHD symptoms and planned versus wanted pregnancies in the present study. Further research will, however, be required to understand the (differential) impacts of ADHD symptoms on these two related but distinct outcomes. Previous research in ADHD has sometimes implicitly assumed that these two outcomes can be treated as interchangeable; however, the present study highlights the importance of making a clear distinction. Indeed, supplementary analyses suggested that the two outcomes were correlated at a level far from unity (*r* = .64 based on a tetrachoric correlation and *r* = .40 based on a point biserial correlation).

We also identified an association between ADHD symptoms during pregnancy and reduced family, friend, and significant other social support. This is consistent with previous evidence that adults with ADHD symptoms experience difficulties in social and romantic relationships. One recent study found that both inattention and impulsivity (but not hyperactivity) symptoms predicted relationship impairment during this period (Eddy et al., 2019). Unfortunately, despite the recognition that relationship issues (especially romantic relationships) can be among the most significant challenges faced by adults with ADHD symptoms, there has been little research into interventions to support this aspect of functioning (Weiss, 2015). For example, most individual interventions for ADHD only address romantic relationship problems indirectly, whereas clinical observation suggests that traditional couple therapy is poorly suited to addressing issues in relationships where one partner is affected by ADHD symptoms (Pera & Robin, 2016). Our results thus provide further impetus for addressing this important gap in relation to adult ADHD support.

### Future Directions

The current study focused on the associations between ADHD symptoms and maternal functioning in pregnancy, as well as neonatal outcomes; however, it is likely that the impact of ADHD symptoms during this time is much broader, and may include impacts on additional health behaviors, planning for the arrival of the baby, and the establishment of adaptive parenting behaviors and family climate. Future research will be beneficial to characterize the full range of effects that ADHD symptoms may have in the perinatal period and how these may impact long-term child development and family well-being. An improved understanding of whether and how to screen for ADHD symptoms in the antenatal period could also have a range of additional benefits. First, identification of maternal ADHD symptoms during pregnancy could facilitate early interventions for parenting behaviors. ADHD symptoms are known to adversely impact parenting and is one mechanism thought to contribute to the intergenerational transmission of symptoms to offspring (Tung et al., 2015). Second, given that parental ADHD is a strong risk factor for ADHD in children, with meta-analytic studies suggesting large proportions of symptom variance (71%–73%) accounted for by genetic factors (Nikolas & Burt, 2010), identification of maternal symptoms in pregnancy could facilitate earlier identification of offspring ADHD. However, further work is required to illuminate how best to screen for ADHD symptoms during pregnancy. It has previously been noted that many current ADHD symptom measures may be suboptimal for identifying symptoms in women. The psychophysiological changes (e.g., mood changes, physical symptoms, tobacco, and alcohol withdrawal) that can occur during pregnancy may, however, further complicate assessment and necessitate tailored measures for the perinatal period.

### Strengths and Limitations

The current study uses a large longitudinal sample from a set of eight culturally diverse countries. However, the majority of the analyses were cross-sectional in nature and we did not have any information on the long-term development of the child nor pre-pregnancy ADHD symptom levels. We were also unable to conduct full diagnostic assessments for ADHD and therefore relied on a screening questionnaire to estimate symptom levels. This also meant that were only able to analyze the impact of ADHD symptoms at a relatively broad level and could not identify the effects of specific types of symptoms (e.g., inattention vs. hyperactivity vs. impulsivity). This will be important to address in future research as there is some preliminary evidence that these domains are differentially related to impairment among pregnant women (Eddy et al., 2019; Jones et al., 2018). We also relied on self-report measures of ADHD; however, it is known that individuals with high levels of ADHD symptoms may sometimes under-report the severity of their difficulties due to positive illusory biases associated with ADHD (e.g., Prevatt et al., 2012). This means that our study could have underestimated the effects of ADHD symptoms due to a reliance on self-reported symptoms. The use of maternal report for birth weight and gestational age will also have reduced the accuracy of preterm and low birth weight classification. Similarly, the internal consistency of the ADHD measure was low, which will have attenuated its associations with the various outcomes evaluated in the present study. Further, we adapted the measure to make it more suitable for pregnant women; however, the version used in the current study has yet to be validated against a gold standard diagnostic measure for ADHD. We, similarly, did not have information about prior diagnosis of ADHD or mental health conditions and only limited (self-reported) information on medication use. Given the nature of the sample (i.e., the contexts in which participants were recruited and the fact that they were pregnant women), it is unlikely that ADHD would have been diagnosed and medicated in our participants (even if participants could meet diagnostic criteria for ADHD), therefore, further research will be needed to clarify the impact of diagnosis and treatment (and is discontinuation) in pregnancy. Similarly, the levels of ADHD symptoms in our sample were overall low. Further complementary research in clinical or high risk samples (or samples enriched for participants with high levels of symptoms) would be beneficial to explore the impact of ADHD symptoms among populations with higher overall levels of ADHD symptoms. It is possible that this would illuminate further associations between ADHD symptoms and maternal and fetal outcomes that were not detectable in the present sample. Finally, it is well-known that ADHD symptoms commonly co-occur with a wide range of other psychiatric issues, including internalizing problems, externalizing problems, and neurodevelopmental conditions (Hollingdale et al., 2020; Tung et al., 2016). In this study we were only able to provide a basic characterization of the associations between ADHD symptoms and issues of relevance for maternal and fetal health; we were unable to tease apart the contributions of ADHD symptoms and commonly co-occurring issues and/or their possible interactions. It will, therefore, be important for future research to explore the impact of ADHD symptoms in the context of other commonly co-occurring psychiatric issues, especially taking into account their possible reciprocal relations and interactions with ADHD symptoms (e.g., Murray, Booth, Obsuth, et al., 2018; Murray et al., 2022).

## Conclusions

ADHD symptoms during pregnancy were associated with higher levels of antenatal stress and depression, lower social support, greater tobacco use, and an increased likelihood that a pregnancy is unwanted and that a birth is premature (though the tobacco use and premature birth associations did not survive correction for multiple comparisons). These preliminary findings underline the importance of further research to improve the assessment of ADHD symptoms in the perinatal period and better understand their impacts on maternal and child outcomes. This information is critical for informing optimal antenatal care for women with ADHD symptoms.

## Supplemental Material

sj-docx-1-jad-10.1177_10870547221105064 – Supplemental material for Associations Between ADHD Symptoms and Maternal and Birth Outcomes: An Exploratory Analysis in a Multi-Country Cohort of Expectant Mothers Click here for additional data file. Supplemental material, sj-docx-1-jad-10.1177_10870547221105064 for Associations Between ADHD Symptoms and Maternal and Birth Outcomes: An Exploratory Analysis in a Multi-Country Cohort of Expectant Mothers by Aja Louise Murray, Diana Taut, Adriana Baban, Chad Lance Hemady, Susan Walker, Joseph Osafo, Siham Sikander, Mark Tomlinson, Stefani Du Toit, Marguerite Marlow, Catherine L. Ward, Asvini Fernando, Bernadette Madrid, Vo Van Thang, Hoang Dinh Tuyen, Michael Dunne, Claire Hughes, Pasco Fearon, Sara Valdebenito and Manuel Eisner in Journal of Attention Disorders

## References

[bibr1-10870547221105064] Abajobir A. A. Maravilla J. C. Alati R. Najman J. M. (2016). A systematic review and meta-analysis of the association between unintended pregnancy and perinatal depression. Journal of Affective Disorders, 192, 56–63. 26707348 10.1016/j.jad.2015.12.008

[bibr2-10870547221105064] Adler L. A. Spencer T. Faraone S. V. Kessler R. C. Howes M. J. Biederman J. Secnik K. (2006). Validity of pilot adult ADHD self-report scale (ASRS) to rate adult ADHD symptoms. Annals of Clinical Psychiatry, 18(3), 145–148. 16923651 10.1080/10401230600801077

[bibr3-10870547221105064] Adler N. E. Epel E. S. Castellazzo G. Ickovics J. R. (2000). Relationship of subjective and objective social status with psychological and physiological functioning: Preliminary data in healthy, White women. Health Psychology, 19(6), 586. 11129362 10.1037//0278-6133.19.6.586

[bibr4-10870547221105064] American Psychiatric Association (2013). Diagnostic and statistical manual of mental disorders (DSM-5^®^). American Psychiatric Publishing.

[bibr5-10870547221105064] Anand S. S. Vasudevan A. Gupta M. Morrison K. Kurpad A. Teo K. K. Srinivasan K. Investigators S. C. S. (2013). Rationale and design of South Asian birth cohort (START): A Canada-India collaborative study. BMC Public Health, 13(1), 79. 23356884 10.1186/1471-2458-13-79 PMC3585827

[bibr6-10870547221105064] Andersson A. Hegvik T.-A. Chen Q. Rosenqvist M. A. Kvalvik L. G. Almqvist C. D’Onofrio B. M. Hartman C. Klungsøyr K. Haavik J. (2020). Attention-deficit/hyperactivity disorder and smoking habits in pregnant women. PLoS ONE, 15(6), e0234561. 32555596 10.1371/journal.pone.0234561 PMC7302708

[bibr7-10870547221105064] Baker A. S. Wales R. Noe O. Gaccione P. Freeman M. P. Cohen L. S. (2020). The course of ADHD during Pregnancy. Journal of Attention Disorders, 26(2), 143–148. 33307923 10.1177/1087054720975864

[bibr8-10870547221105064] Baker L. Prevatt F. Proctor B. (2012). Drug and alcohol use in college students with and without ADHD. Journal of Attention Disorders, 16(3), 255–263. 21828360 10.1177/1087054711416314

[bibr9-10870547221105064] Bale T. L. (2015). Epigenetic and transgenerational reprogramming of brain development. Nature Reviews Neuroscience, 16(6), 332–344. 25921815 10.1038/nrn3818 PMC7064155

[bibr10-10870547221105064] Bergman K. Sarkar P. O’Connor T. G. Modi N. Glover V. (2007). Maternal stress during pregnancy predicts cognitive ability and fearfulness in infancy. Journal of the American Academy of Child & Adolescent Psychiatry, 46(11), 1454–1463. 18049295 10.1097/chi.0b013e31814a62f6

[bibr11-10870547221105064] Betts K. S. Williams G. M. Najman J. M. Alati R. (2014). Maternal depressive, anxious, and stress symptoms during pregnancy predict internalizing problems in adolescence. Depression and Anxiety, 31(1), 9–18. 24395339 10.1002/da.22210

[bibr12-10870547221105064] Biederman J. Ball S. W. Monuteaux M. C. Mick E. Spencer T. J. McCreary M. Cote M. Faraone S. V. (2008). New insights into the comorbidity between ADHD and major depression in adolescent and young adult females. Journal of the American Academy of Child & Adolescent Psychiatry, 47(4), 426–434. 18388760 10.1097/CHI.0b013e31816429d3

[bibr13-10870547221105064] Biederman J. Faraone S. Spencer T. Mick E. Monuteaux M. Aleardi M. (2006). Functional impairments in adults with self-reports of diagnosed ADHD. Journal of Clinical Psychiatry, 67(4), 524–540. 16669717 10.4088/jcp.v67n0403

[bibr14-10870547221105064] Bruner M. R. Kuryluk A. D. Whitton S. W. (2015). Attention-deficit/hyperactivity disorder symptom levels and romantic relationship quality in college students. Journal of American College Health, 63(2), 98–108. 25350392 10.1080/07448481.2014.975717

[bibr15-10870547221105064] Cohen S. (1988). Perceived stress in a probability sample of the United States. In Spacapan S. Oskamp S. (Eds.), The social psychology of health (pp. 31–67). SAGE.

[bibr16-10870547221105064] Committee on Substance Abuse and Committee on Children With Disabilities. (2000). Fetal alcohol syndrome and alcohol-related neurodevelopmental disorders. Pediatrics, 106(2), 358–361. 10920168

[bibr17-10870547221105064] Eddy L. D. Jones H. A. Snipes D. Karjane N. Svikis D. (2019). Associations between ADHD symptoms and occupational, interpersonal, and daily life impairments among pregnant women. Journal of Attention Disorders, 23(9), 976–984. 28043206 10.1177/1087054716685839

[bibr18-10870547221105064] Eisner N. L. Murray A. L. Eisner M. Ribeaud D. (2018). A practical guide to the analysis of non-response and attrition in longitudinal research using a real data example. International Journal of Behavioral Development, 43(1), 24–34.

[bibr19-10870547221105064] Fayyad J. Sampson N. A. Hwang I. Adamowski T. Aguilar-Gaxiola S. Al-Hamzawi A. Andrade L. H. Borges G. de Girolamo G. Florescu S. (2017). The descriptive epidemiology of DSM-IV adult ADHD in the world health organization world mental health surveys. ADHD Attention Deficit and Hyperactivity Disorders, 9(1), 47–65. 27866355 10.1007/s12402-016-0208-3 PMC5325787

[bibr20-10870547221105064] Fite P. J. Evans S. C. Cooley J. L. Rubens S. L. (2014). Further evaluation of associations between attention-deficit/hyperactivity and oppositional defiant disorder symptoms and bullying-victimization in adolescence. Child Psychiatry & Human Development, 45(1), 32–41. 23516013 10.1007/s10578-013-0376-8

[bibr21-10870547221105064] Flower A. Shawe J. Stephenson J. Doyle P. (2013). Pregnancy planning, smoking behaviour during pregnancy, and neonatal outcome: UK millennium cohort study. BMC Pregnancy and Childbirth, 13(1), 238. 24354748 10.1186/1471-2393-13-238 PMC3878353

[bibr22-10870547221105064] Freeman M. P. (2014). ADHD and pregnancy. American Journal of Psychiatry, 171(7), 723–728. 24980168 10.1176/appi.ajp.2013.13050680

[bibr23-10870547221105064] Gershon J. Gershon J. (2002). A meta-analytic review of gender differences in ADHD. Journal of Attention Disorders, 5(3), 143–154. 11911007 10.1177/108705470200500302

[bibr24-10870547221105064] Giesbrecht G. F. Poole J. C. Letourneau N. Campbell T. Kaplan B. J. , & APrON Study Team. (2013). The buffering effect of social support on hypothalamic-pituitary-adrenal axis function during pregnancy. Psychosomatic Medicine, 75(9), 856–862. 24163383 10.1097/PSY.0000000000000004

[bibr25-10870547221105064] Goossens J. Van Den Branden Y. Van der Sluys L. Delbaere I. Van Hecke A. Verhaeghe S. Beeckman D. (2016). The prevalence of unplanned pregnancy ending in birth, associated factors, and health outcomes. Human Reproduction, 31(12), 2821–2833. 27798048 10.1093/humrep/dew266

[bibr26-10870547221105064] Gou X. Wang Y. Tang Y. Qu Y. Tang J. Shi J. Xiao D. Mu D. (2019). Association of maternal prenatal acetaminophen use with the risk of attention deficit/hyperactivity disorder in offspring: A meta-analysis. Australian & New Zealand Journal of Psychiatry, 53(3), 195–206. 30654621 10.1177/0004867418823276

[bibr27-10870547221105064] Groen-Blokhuis M. M. Middeldorp C. M. Kan K.-J. Abdellaoui A. Van Beijsterveldt C. E. Ehli E. A. Davies G. E. Scheet P. A. Xiao X. Hudziak J. J. (2014). Attention-deficit/hyperactivity disorder polygenic risk scores predict attention problems in a population-based sample of children. Journal of the American Academy of Child & Adolescent Psychiatry, 53(10), 1123–1129. 25245356 10.1016/j.jaac.2014.06.014

[bibr28-10870547221105064] Hetherington E. Doktorchik C. Premji S. S. McDonald S. W. Tough S. C. Sauve R. S. (2015). Preterm birth and social support during pregnancy: A systematic review and meta-analysis. Paediatric and Perinatal Epidemiology, 29(6), 523–535. 26332279 10.1111/ppe.12225

[bibr29-10870547221105064] Hollingdale J. Woodhouse E. Young S. Fridman A. Mandy W. (2020). Autistic spectrum disorder symptoms in children and adolescents with attention-deficit/hyperactivity disorder: A meta-analytical review. Psychological Medicine, 50(13), 2240–2253. 31530292 10.1017/S0033291719002368

[bibr30-10870547221105064] Jarrett M. A. (2016). Attention-deficit/hyperactivity disorder (ADHD) symptoms, anxiety symptoms, and executive functioning in emerging adults. Psychological Assessment, 28(2), 245. 26121381 10.1037/pas0000190

[bibr31-10870547221105064] Jones H. A. Eddy L. D. Rabinovitch A. E. Snipes D. J. Wilson S. A. Parks A. M. Karjane N. W. Svikis D. S. (2018). Attention-deficit/hyperactivity disorder symptom clusters differentially predict prenatal health behaviors in pregnant women. Journal of Clinical Psychology, 74(4), 665–679. 28945932 10.1002/jclp.22538

[bibr32-10870547221105064] Kessler R. C. Adler L. Ames M. Barkley R. A. Birnbaum H. Greenberg P. Johnston J. A. Spencer T. Üstün T. B. (2005). The prevalence and effects of adult attention deficit/hyperactivity disorder on work performance in a nationally representative sample of workers. Journal of Occupational and Environmental Medicine, 47(6), 565–572. 15951716 10.1097/01.jom.0000166863.33541.39

[bibr33-10870547221105064] Kessler R. C. Adler L. Barkley R. Biederman J. Conners C. K. Demler O. Faraone S. V. Greenhill L. L. Howes M. J. Secnik K. (2006). The prevalence and correlates of adult ADHD in the United States: Results from the National Comorbidity Survey Replication. American Journal of Psychiatry, 163(4), 716–723. 16585449 10.1176/appi.ajp.163.4.716 PMC2859678

[bibr34-10870547221105064] Kittel-Schneider S. Quednow B. B. Leutritz A. L. McNeill R. V. Reif A. (2021). Parental ADHD in pregnancy and the postpartum period—A systematic review. Neuroscience & Biobehavioral Reviews. 10.1016/j.neubiorev.2021.01.002 33516734

[bibr35-10870547221105064] Knopik V. S. Marceau K. Bidwell L. C. Palmer R. H. Smith T. F. Todorov A. Evans A. S. Heath A. C. (2016). Smoking during pregnancy and ADHD risk: A genetically informed, multiple-rater approach. American Journal of Medical Genetics Part B: Neuropsychiatric Genetics, 171(7), 971–981. 10.1002/ajmg.b.32421 PMC4958030 26799787

[bibr36-10870547221105064] Kroenke K. Spitzer R. L. Williams J. B. (2001). The PHQ-9: Validity of a brief depression severity measure. Journal of General Internal Medicine, 16(9), 606–613. 11556941 10.1046/j.1525-1497.2001.016009606.x PMC1495268

[bibr37-10870547221105064] Krone B. Newcorn J. H. (2015). Comorbidity of ADHD and anxiety disorders. In Adler L. A. Spencer T. J. Wilens T. E. (Eds.), Attention-deficit hyperactivity disorder in adults and children (pp. 98–110). Cambridge University Press.

[bibr38-10870547221105064] Kuznetsova A. Brockhoff P. B. Christensen R. H. (2017). lmerTest package: Tests in linear mixed effects models. Journal of Statistical Software, 82(13), 1–26.

[bibr39-10870547221105064] Laplante D. P. Barr R. G. Brunet A. Du Fort G. G. Meaney M. L. Saucier J.-F. Zelazo P. R. King S. (2004). Stress during pregnancy affects general intellectual and language functioning in human toddlers. Pediatric Research, 56(3), 400–410. 15240860 10.1203/01.PDR.0000136281.34035.44

[bibr40-10870547221105064] Lee S. S. Humphreys K. L. Flory K. Liu R. Glass K. (2011). Prospective association of childhood attention-deficit/hyperactivity disorder (ADHD) and substance use and abuse/dependence: A meta-analytic review. Clinical Psychology Review, 31(3), 328–341. 21382538 10.1016/j.cpr.2011.01.006 PMC3180912

[bibr41-10870547221105064] Luke S. G. (2017). Evaluating significance in linear mixed-effects models in R. Behavior Research Methods, 49(4), 1494–1502. 27620283 10.3758/s13428-016-0809-y

[bibr42-10870547221105064] Martinez-Torteya C. Bogat G. A. Levendosky A. A. Von Eye A. (2016). The influence of prenatal intimate partner violence exposure on hypothalamic–pituitary–adrenal axis reactivity and childhood internalizing and externalizing symptoms. Development and Psychopathology, 28(1), 55–72. 25851078 10.1017/S0954579415000280

[bibr43-10870547221105064] McKee T. E. (2017). Peer relationships in undergraduates with ADHD symptomatology: Selection and quality of friendships. Journal of Attention Disorders, 21(12), 1020–1029. 25359763 10.1177/1087054714554934

[bibr44-10870547221105064] Murray A. L. Booth T. Obsuth I. Zirk-Sadowski J. Eisner M. Ribeaud D. (2018). Testing the exacerbation and attenuation hypotheses of the role of anxiety in the relation between ADHD and reactive/proactive aggression: A 10-year longitudinal study. Psychiatry Research, 269, 585–592. 30205351 10.1016/j.psychres.2018.08.120

[bibr45-10870547221105064] Murray A. L. Caye A. McKenzie K. Auyeung B. Murray G. Ribeaud D. Freeston M. Eisner M. (2022). Reciprocal developmental relations between ADHD and anxiety in adolescence: A within-person longitudinal analysis of commonly co-occurring symptoms. Journal of Attention Disorders, 26(1), 109–118. 32172640 10.1177/1087054720908333

[bibr46-10870547221105064] Murray A. L. Eisner M. Obsuth I. Ribeaud D. (2017). No evidence that substance use causes ADHD symptoms in adolescence. Journal of Drug Issues, 47(3), 405–410.

[bibr47-10870547221105064] Murray A. L. Wong S.-C. Obsuth I. Rhodes S. Eisner M. Ribeaud D. (2020). An ecological momentary assessment study of the role of emotional dysregulation in co-occurring ADHD and internalising symptoms in adulthood. Journal of Affective Disorders, 281, 708–713. 33234281 10.1016/j.jad.2020.11.086

[bibr48-10870547221105064] Nikolas M. A. Burt S. A. (2010). Genetic and environmental influences on ADHD symptom dimensions of inattention and hyperactivity: A meta-analysis. Journal of Abnormal Psychology, 119(1), 1. 20141238 10.1037/a0018010

[bibr49-10870547221105064] Ninowski J. E. Mash E. J. Benzies K. M. (2007). Symptoms of attention-deficit/hyperactivity disorder in first-time expectant women: Relations with parenting cognitions and behaviors. Infant Mental Health Journal, 28(1), 54–75. 28640382 10.1002/imhj.20122

[bibr50-10870547221105064] Oliver M. L. Han K. Bos A. J. Backs R. W. (2015). The relationship between ADHD symptoms and driving behavior in college students: The mediating effects of negative emotions and emotion control. Transportation Research Part F: Traffic Psychology and Behaviour, 30, 14–21.

[bibr51-10870547221105064] Owens E. B. Hinshaw S. P. (2020). Adolescent mediators of unplanned pregnancy among women with and without childhood ADHD. Journal of Clinical Child & Adolescent Psychology, 49(2), 229–238. 30689435 10.1080/15374416.2018.1547970 PMC6661209

[bibr52-10870547221105064] Pera G. Robin A. L. (2016). Adult adhd-focused couple therapy: Clinical interventions. Routledge.

[bibr53-10870547221105064] Prevatt F. Proctor B. Best L. Baker L. Van Walker J. Taylor N. W. (2012). The positive illusory bias: Does it explain self-evaluations in college students with ADHD? Journal of Attention Disorders, 16(3), 235–243. 21289235 10.1177/1087054710392538

[bibr54-10870547221105064] Rini C. Schetter C. D. Hobel C. J. Glynn L. M. Sandman C. A. (2006). Effective social support: Antecedents and consequences of partner support during pregnancy. Personal Relationships, 13(2), 207–229.

[bibr55-10870547221105064] Rohde P. Kahler C. W. Lewinsohn P. M. Brown R. A. (2004). Psychiatric disorders, familial factors, and cigarette smoking: II. Associations with progression to daily smoking. Nicotine & Tobacco Research, 6(1), 119–132. 14982696 10.1080/14622200310001656948

[bibr56-10870547221105064] Ruiz-Goikoetxea M. Cortese S. Aznarez-Sanado M. Magallon S. Zallo N. A. Luis E. O. de Castro-Manglano P. Soutullo C. Arrondo G. (2018). Risk of unintentional injuries in children and adolescents with ADHD and the impact of ADHD medications: A systematic review and meta-analysis. Neuroscience & Biobehavioral Reviews, 84, 63–71. 29162520 10.1016/j.neubiorev.2017.11.007

[bibr57-10870547221105064] Schmidt D. Seehagen S. Vocks S. Schneider S. Teismann T. (2016). Predictive importance of antenatal depressive rumination and worrying for maternal–Foetal attachment and maternal well-being. Cognitive Therapy and Research, 40(4), 565–576.

[bibr58-10870547221105064] Sosnowski D. W. Booth C. York T. P. Amstadter A. B. Kliewer W. (2018). Maternal prenatal stress and infant DNA methylation: A systematic review. Developmental Psychobiology, 60(2), 127–139. 29344930 10.1002/dev.21604

[bibr59-10870547221105064] Tremblay R. E. Loeber R. Gagnon C. Charlebois P. Larivee S. LeBlanc M. (1991). Disruptive boys with stable and unstable high fighting behavior patterns during junior elementary school. Journal of Abnormal Child Psychology, 19(3), 285–300. 1865046 10.1007/BF00911232

[bibr60-10870547221105064] Treur J. L. Demontis D. Smith G. D. Sallis H. Richardson T. G. Wiers R. W. Børglum A. D. Verweij K. J. Munafò M. R. (2019). Investigating causality between liability to ADHD and substance use, and liability to substance use and ADHD risk, using Mendelian randomization. Addiction Biology, 26(1), e12849. 31733098 10.1111/adb.12849 PMC7228854

[bibr61-10870547221105064] Tung I. Brammer W. A. Li J. J. Lee S. S. (2015). Parenting behavior mediates the intergenerational association of parent and child offspring ADHD symptoms. Journal of Clinical Child & Adolescent Psychology, 44(5), 787–799. 24926775 10.1080/15374416.2014.913250 PMC4755584

[bibr62-10870547221105064] Tung I. Li J. J. Meza J. I. Jezior K. L. Kianmahd J. S. Hentschel P. G. O’Neil P. M. Lee S. S. (2016). Patterns of comorbidity among girls with ADHD: A meta-analysis. Pediatrics, 138(4), e20160430. 27694280 10.1542/peds.2016-0430 PMC9923580

[bibr63-10870547221105064] Valdebenito S. Murray A. Hughes C. Băban A. Fernando A. D. Madrid B. J. Ward C. Osafo J. Dunne M. Sikander S. (2020). Evidence for better lives study: A comparative birth-cohort study on child exposure to violence and other adversities in eight low-and middle-income countries-foundational research (study protocol). BMJ Open, 10(10), e034986. 10.1136/bmjopen-2019-034986 PMC7552842 33039982

[bibr64-10870547221105064] Vythilingum B. Roos A. Faure S. C. Geerts L. Stein D. J. (2012). Risk factors for substance use in pregnant women in South Africa. South African Medical Journal, 102(11), 853–854. 10.7196/samj.5019 23116742

[bibr65-10870547221105064] Weiss M. (2015). Functional impairment in ADHD. In Adler L. A. Spencer T. J. Wilens T. E. (Eds.), Attentiondeficit hyperactivity disorder in adults and children (pp. 42–52). Cambridge University Press.

[bibr66-10870547221105064] WHO ASSIST Working Group. (2002). The alcohol, smoking and substance involvement screening test (ASSIST): Development, reliability and feasibility. Addiction, 97(9), 1183–1194. 12199834 10.1046/j.1360-0443.2002.00185.x

[bibr67-10870547221105064] Williamson D. Johnston C. (2015). Gender differences in adults with attention-deficit/hyperactivity disorder: A narrative review. Clinical Psychology Review, 40, 15–27. 26046624 10.1016/j.cpr.2015.05.005

[bibr68-10870547221105064] Wymbs B. T. Dawson A. E. Suhr J. A. Bunford N. Gidycz C. A. (2017). ADHD symptoms as risk factors for intimate partner violence perpetration and victimization. Journal of Interpersonal Violence, 32(5), 659–681. 26025345 10.1177/0886260515586371

[bibr69-10870547221105064] Yanikkerem E. Ay S. Piro N. (2013). Planned and unplanned pregnancy: Effects on health practice and depression during pregnancy. Journal of Obstetrics and Gynaecology Research, 39(1), 180–187. 22889435 10.1111/j.1447-0756.2012.01958.x

[bibr70-10870547221105064] Zimet G. D. Dahlem N. W. Zimet S. G. Farley G. K. (1988). The multidimensional scale of perceived social support. Journal of Personality Assessment, 52(1), 30–41. 10.1080/00223891.1990.9674095 2280326

[bibr71-10870547221105064] Zimet G. D. Powell S. S. Farley G. K. Werkman S. Berkoff K. A. (1990). Psychometric characteristics of the multidimensional scale of perceived social support. Journal of Personality Assessment, 55(3–4), 610–617. 2280326 10.1080/00223891.1990.9674095

