# Supplementary material for: Associations Between ADHD Symptoms and Maternal and Birth Outcomes: An Exploratory Analysis in a Multi-Country Cohort of Expectant Mothers
Source: J Atten Disord. 2022 Jul 11;26(14):1882–94. doi: 10.1177/10870547221105064 (PMC9597155; doi:10.1177/10870547221105064)
Supplement: sj-docx-1-jad-10.1177_10870547221105064 – Supplemental material for Associations Between ADHD Symptoms and Maternal and Birth Outcomes: An Exploratory Analysis in a Multi-Country Cohort of Expectant Mothers [file sj-docx-1-jad-10.1177_10870547221105064.docx]

**Supplementary Materials**

**Table S1: ADHD item wordings**

| The following statements describe how people sometimes behave or feel. Please tell us how well each statement describes how you have felt and conducted yourself over the past 6 months. That is from ‘month’ till now. Did you feel that way never, rarely, sometimes, often or always? Remember, there are no right or wrong answers only you can say how you feel. |
| --- |

|  | **Never** | **Rarely** | **Sometimes** | **Often** | **Always** |
| --- | --- | --- | --- | --- | --- |
| 7.38 How often do you have difficulty concentrating on what people say to you, even when they are speaking to you directly? | 1 | 2 | 3 | 4 | 5 |
| 7.39 How often do you leave your seat in situations in which you are expected to remain seated? | 1 | 2 | 3 | 4 | 5 |
| 7.40 How often do you have difficulty unwinding and relaxing when you have time to yourself? | 1 | 2 | 3 | 4 | 5 |
| 7.41 How often do you put things off until the last minute? | 1 | 2 | 3 | 4 | 5 |
| 7.42 How often do you depend on others to keep your life in order and attend to details? | 1 | 2 | 3 | 4 | 5 |
